# Green Biased Technical Change in Terms of Industrial Water Resources in China’s Yangtze River Economic Belt

**DOI:** 10.3390/ijerph17082789

**Published:** 2020-04-17

**Authors:** Xiyue Zhang, Fangcheng Sun, Huaizu Wang, Yi Qu

**Affiliations:** 1 Research Center for Economy of Upper Reaches of the Yangtze River, Chongqing Technology and Business University, Chongqing 400067, China; zhangxy@cque.edu.cn (X.Z.); fcsun28@ctbu.edu.cn (F.S.); 2 College of Economics and Business Administration, Chongqing University of Education, Chongqing 400067, China; 3 College of Intellectual Property, Chongqing University of Technology, Chongqing 400054, China; 4 Newcastle Business School, Northumbria University, Newcastle upon Tyne NE1 8ST, UK; yi.qu@northumbria.ac.uk

**Keywords:** green biased technical change, green productivity, industrial water resources, Yangtze River Economic Belt

## Abstract

As a significant ecological corridor from west to east across China, the Yangtze River Economical Belt (YREB) is in great need of green development and transformation. Rather than only focusing on the overall growth of green productivity, it is important to identify whether the technical change is biased towards economic performance or green performance in promoting green productivity. By employing the biased technical change theory and Malmquist index decomposition method, we analyze the green biased technical change in terms of industrial water resources in YREB at the output side and the input side respectively. We find that the green biased technical change varies during 2006–2015 at both the input side and output side in YREB. At the input side, water-saving biased technical change is generally dominant compared to water-using biased technical change during 2006–2015, presenting the substitution effects of non-water production factors. At the output side, the economy-growth biased technical change is the main force to promote green productivity, whereas the role of water-conservation biased technical change is insufficient. The green performance at the output side needs to be strengthened compared to the economic performance in YREB. A series of water-related environmental policies introduced in China since 2008 have promoted the green biased technical change both at the input side and the output side in YREB, but the policy effects at the output side is still inadequate compared to that at the input side. The technological innovation in sewage treatment and control need to catch up with the economic growth in YREB. Our research gives insights to enable a deeper understanding of the green biased technical change in YREB and will benefit more focused policy-making of green innovation.

## 1. Introduction

**Figure 1:**
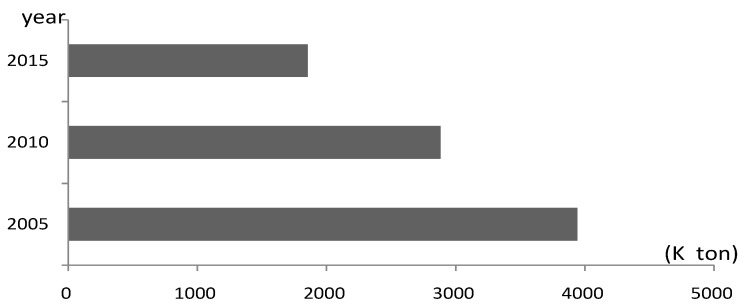
Total chemical oxygen demand (COD) discharge in industrial sewage of top 10 water-intensive industries in China. Data sources: China Environmental Statistical Yearbook (2005–2015).

**Figure 2:**
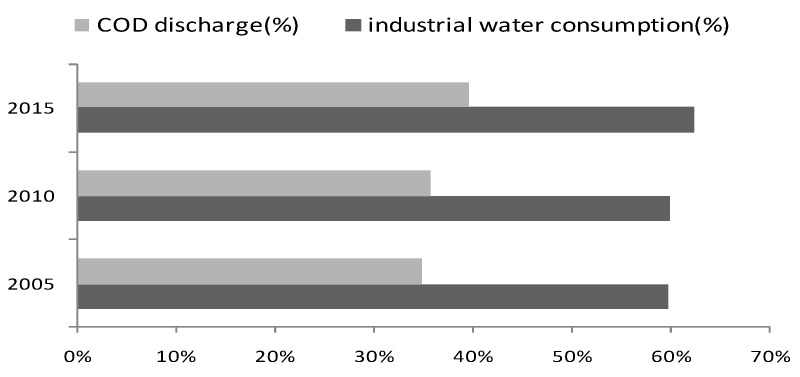
Proportion of total industrial water consumption and COD discharge in the Yangtze River Economic Belt (YREB) compared to nationwide. Data sources: China Environmental Statistical Yearbook (2005–2015).

The Yangtze River Economic Belt (hereafter, YREB) is one of the most active areas in economic and social development in China, and is also an important ecological corridor from west to east across China. In recent years, China has exerted great efforts in environmental protection and ecological civilization construction. The quality of water resources in YREB has improved steadily, but the ecological system in YREB is still weak and the risk of pollution is still high. Water protection in YREB still has a long way to go especially considering the balance of economic development and water protection. Since 2008, the Chinese government has issued a series of water environmental laws and policies such as the “Amendment of China Water Pollution Prevention and Control Law”, “Opinions on Implementing the Strictest Water Resources Management System” and the “Water Pollution Prevention and Control Action Plan”, which have imposed the strictest requirements on water conservation in China. Under the influence of new water-related regulations and policies, the discharge of pollutants from industrial sewage in China has continued to decrease. Figure 1 reveals the total chemical oxygen demand (COD) discharge of sewage in China’s top 10 water-intensive industries (including industries of textiles, paper-making, metal smelting, chemical, power generation, etc.). According to Figure 1, the COD discharge of sewage in the top 10 water-intensive industries across China in 2015 has dropped over 50% compared to that in 2005. However, as the overall COD discharge is still large, the task of controlling industrial sewage and related pollutants remains severe. YREB is a cluster area for China’s water-intensive industries. For example, according to China’s industry statistical yearbooks, since 2005, the yearly output value of chemical fiber products in YREB has accounted for more than 70% of the total amount in China. This has brought tremendous pressure on industrial water using and sewage discharge in YREB. According to Figure 2, in each year of 2005, 2010 and 2015, the amount of industrial water consumption in YREB has accounted for over 60% of the total amount in China, and the amount of industrial COD discharge has accounted for over 30% of the total amount in China. It is urgent to promote green productivity and green transformation development in terms of industrial water resources in YREB.

A program released by the Chinese government in 2016 called “innovation-driven industrial transformation and upgrading in YREB” has emphasized the important role of innovation strategy for the green transformation and development of YREB. Although economic performance and green performance are usually in contradiction, the innovation-driven strategy can achieve synergy between economic performance and green performance. Green TFP (total factor productivity) is an important indicator for measuring the coordinated development of economic growth and environmental protection. However, there exist different technological directions or bias for improving green TFP. Some regions may prefer to choose neutral or less green technologies as long as they can promote the rapid growth of economy. Even if the green performance does not increase much, the overall green productivity can be promoted by the economic performance. While other regions may prefer to choose more green technologies, which can vigorously improve the green performance. Even if the economic growth is slow, the overall green productivity can be promoted by the improvement of green performance. For YREB, although the economic performance and green performance are both important, which one is the main driver for green productivity growth may vary in different regions and at different times. For example, some areas with severe environmental problems should be strongly encouraged to adopt the environmentally biased technical change mode. Rather than only focus on the overall growth of green productivity, it is necessary to distinguish whether technical change is biased towards economic performance or green performance in promoting green productivity. By employing the biased technical change theory and Malmquist index decomposition method, we analyze the green biased technical change in terms of industrial water resources in YREB from the output side and the input side respectively. Our research will benefit more focused policy-making of green innovation.

## 2. Literature Review 

Technical change is biased when it increases the marginal product of one production factor more than another [1]. The concept of biased technical change originates from early “induced innovation” literature proposed first by Hicks [2], who points out that technical change tends to save more expensive production factors. Acemoglu [1,3] develops the biased technical change theory from the induced innovation theory by laying out the micro-foundations in the framework of endogenous technical change models, and making the biased technical change theory widely used, especially in explaining the skill wage with the skill-biased technical change. Simultaneously, the green biased technical change and its impact on environmental protection and economical growth is getting more and more attention. Popp [4,5] documents the impact of energy prices on patents for energy-saving innovations, finding that higher energy prices are associated with a significant increase in energy-saving innovations, and the ignorance of biased technical change might overstate the costs of environmental regulation. Acemoglu et al. [6] introduce biased technical change under an endogenous growth model with environmental constraints, suggesting that the combination of research subsidies and carbon taxes can be an optimal strategy to redirect technical change toward cleaner technologies. Acemoglu et al. [7] further develop an endogenous growth model in which clean and dirty technologies compete in production, finding that if dirty technologies are more advanced, the transition to clean technology can be difficult. Calel et al. [8] find that the EU emissions trading scheme has increased low-carbon innovation among regulated firms by as much as 10%, while not crowding out patenting for other technologies. Aghion et al. [9] find a sizable impact of carbon taxes on the direction of innovation in the automobile industry. Some researches study the green biased technical change in China. Among them, Wang et al. [10] estimate China’s biased technical change under environmental constraints from 2004 to 2015, finding that although the rapid accumulation of capital leads to technical progress that is biased toward capital, technical progress in the labor bias can significantly increase green total factor productivity. Jiang et al. [11] split the industry performance into economic and environmental dimensions, finding that technical and scale inefficiencies are relatively higher for environmental sub-technology compared to the economic sub-technology in China. Peng et al. [12] find that the output-biased technical change is the significant contributor to the technical change in the Chinese energy industry from 2006 to 2016.

Our research is also closely related to literature on the green total factor productivity in terms of water resources. With the growth of the global population and economic development, the paradox between water-using and water-protection has been increasing considerably. The World Economic Forum [13] has identified the water supply crises as one of the top three global risks. More and more researchers are focusing on water-using and water-treatment efficiency. The related research includes water efficiency in industry [14,15], agriculture [16,17], domestic water [18,19] and the comprehensive water efficiency including both industrial and domestic water [20,21]. Among them, research into water efficiency and protection in industry has gained the most attention, especially in newly industrialized countries. According to the forecast of OECD (Organization for Economic Cooperation and Development) [22], global manufacturing water demand is projected to increase fourfold during 2000–2050. The synergy among industrial economic growth, industrial water demand and water protection plays an important role in sustainable industrial economic development, which is of more significance for China due to its rapid industrial economic growth in the last three decades under the pressure of limited water resources. The industrial green water productivity is a comprehensive productivity indicator that considers both industrial economic growth and industrial water related activities. According to the definition of total-factor water efficiency by Hu et al. [23], the industrial green water productivity is defined as the industrial productivity considering the industrial water input and the non-expected output of wastewater related pollutant discharge, as well as the conventional production inputs and outputs. Among the literature in Chinese industrial green water productivity, some scholars have studied the time and space distribution of green water productivity across China [14,15]. Some research is relevant to the two-stage productivity of industrial water consumption and industrial wastewater treatment [24]. Technical change is one of the main drivers to influence productivity, which can be estimated by decomposing the total factor productivity [25]. Moro et al. [26] find a rapid Chinese technological catching up of water innovation in the last three decades using patent data. Yet the properties of technical change in terms of water resources and their influence on the industrial green water productivity need to be further studied. Jin et al. [27] show that the impact of technological innovation on green industrial water productivity is different spatially in China. As the Yangtze River Economic Belt (YREB) is an important ecological corridor from west to east across China, some studies focus on the green productivity and technical change in YREB. Xing et al. [28] explore the total factor ecological efficiency in YREB based on a proposed Shephard energy distance function. Peng et al. [29] find out the environmental governance efficiency for the Yangtze River urban agglomeration is basically driven by technical progress. Wang and Yang [30] explore the mechanism of three innovation factors including innovative human capital, R&D fund, and fixed assets to the technological innovation performance in the Yangtze River Delta region. Our research focuses on the green bias of technical change in terms of industrial water resources in YREB using a non-parametric approach, which has received less scrutiny in the existing research.

There are generally two types of methods for measuring biased technology change in the existing research. One is the parameter method, by setting different production function models and solving the parameters, and then calculating the index of biased technical change [1,31]. Parametric methods can conduct conventional tests of hypotheses, and can accommodate the effects of data noise, but they need to assume specific forms of production functions. The second one is the non-parametric approach employing data envelopment analysis method (DEA) or Malmquist index decomposition method, which decomposes the technical change index into the input-biased technical change index, the output-biased technical change index and the neutral technical change index [32,33,34]. The second method can deal with multiple inputs and multiple outputs simultaneously without giving specific production function forms, which fully takes into account the diversity of production technology in the economy. Weber and Domazlicky [33] and Barros and Weber [35] illustrate the criteria to identify which input or output is favored in the biased technical change with input-based Malmquist index. Färe et al. [36] demonstrate the criteria for identifying the direction of input bias in the biased technical change with output-based Malmquist index. There is still a lack of criteria for identifying the direction of output bias in the biased technical change with the output-based Malmquist index method according to the existing research literature. Given that the output-based Malmquist index is widely used in the study of productivity growth and technical change [37,38,39], we demonstrate the criteria for identifying the directions of both the output and input bias in the biased technical change with the output-based Malmquist index method. We employ the Shephard radial distance function to calculate the Malmquist index because it is the analytical basis in constructing the identifying criteria for the biased technical change with the Malmquist index method [33]. Some scholars try to use this kind of identifying the criteria with non-radial distance functions. For example, Li et al. [40] use the directional distance function with undesired output to analyze the green biased technical progress in terms of water resources across China on the basis of Weber and Domazlicky’s research [33]. The directional distance function with an undesired output is a non-radial distance function. Although the non-radial distance function is employed to calculate the Malmquist index, a radial distance function is actually employed to derive the identifying criteria for biased technical change based on that Malmquist index in their research. This causes a contradiction in their method. According to the present literature, whether the identifying criteria for biased technical change based on the Weber and Domazlicky’s method [33] applies to non-radial distance function has not yet been sufficiently proven. This paper employs the Shephard radial distance function to construct the identifying criteria for biased technical change with the output-based Malmquist index, and then uses this framework and the biased technical change theory to study the green biased technical change in YREB in terms of water resources.

The growth of green productivity can be driven by the economic performance as well as the green performance. Some regions can realize the growth of green productivity mainly by higher economic performance, which can offset the relatively lower green performance. On the contrary, some other regions can realize the growth of green productivity mainly by higher green performance, which can offset the relatively lower economic performance. The different dominance between economic performance and green performance depends on the different bias of technical change. The technical change can be divided into two modes including economically biased technical change and green biased technical change. In the economically biased technical change mode, neutral or less green technologies are more preferred as long as they can promote the rapid growth of economy to offset the relatively low green performance, while in the green biased technical change mode, more green technologies are preferred, which can vigorously improve the green performance. By analyzing the two different kinds of technical change, we can further reveal the characteristics of green bias in the process of green economic transformation rather than only focus on the overall growth of green productivity. In our research, we will discuss the green biased technical change in terms of industrial water resources in YREB. Has the technical change contributed to the green productivity growth in YREB in terms of industrial water resources? Is the technical change biased to economic performance or water-related green performance in YREB? What is the difference in the green biased technical change at the input side and the output side respectively in YREB? We will employ the biased technical change theory and Malmquist index decomposition method to explore these questions.

The structure of this paper is organized as follows. Section 2 provides a summary of current related research, including the research of biased technical change and the green total factor productivity in terms of water resources. Section 3 illustrates the measuring methods, variable indicators and data resources, as well as the demonstration of identifying criteria for biased technical change. Section 4 presents and analyzes the results of green productivity and the directions of biased technical change in terms of water resources in YREB. Conclusions and policy implications are presented in Section 5.

## 3. Model and Data

### 3.1. Model for Measuring Biased Technical Change

The DEA method has been widely used to estimate the Shephard output distance function [41], which can serve as a measure of output technical efficiency [42] and equals the ratio of actual outputs of a DMU (decision-making unit) to the potentially optimal outputs of DMUs in the production frontier holding inputs constant. The output technical efficiency for DMU “*k*” can be obtained by solving the following linear programming problem,

(1) $$D_{O}{}^{t}(x_{k}{}^{t},y_{k}{}^{t})=\min\left\{\theta :\sum_{i=1}^{R}\lambda_{i}^{t}x_{ni}^{t}\leq x_{nk}^{t},n=1\cdots N;\sum_{i=1}^{R}\lambda_{i}^{t}y_{mi}^{t}\geq \theta^{-1}y_{mk}^{t},m=1\cdots M;\lambda_{i}^{t}\geq 0,i=1\cdots R\right\}$$

where $D_{O}{}^{t}(x_{k}{}^{t},y_{k}{}^{t})$ is the Shephard output distance function or the output technical efficiency for DMU “*k*” among the *R* DMUs when constant returns to scale holds. $x_{n}^{t}$ represents the *N* non-negative inputs in period *t*, and $y_{m}^{t}$ represents the *M* non-negative outputs produced in period *t*. $\lambda_{i}^{t}$ are the intensity variables in period *t*, which are non-negative.

Following Färe et al. [25], the output technical efficiency growth can be estimated using the output-based Malmquist index (hereafter MI) as shown in Formula (2). This index can be decomposed into two indices measuring efficiency change (hereafter EC) and technical change (hereafter TC). The efficiency change measures the ‘‘catching up” to the production frontier, reflecting the change of organizational management ability, while the technical change measures the shift of the production frontier from one period to another, reflecting the level of technical progress. As the product of EC and TC, the MI index reflects the overall technical efficiency growth of each DMU. According to Färe et al. [25], the output-based Malmquist index and the decomposition indices take the following form

(2) $$\begin{array}{l} {\mathrm{MI}}_{o}^{t,t+1}(x^{t},y^{t})={\left(\frac{D_{o}{}^{t+1}(x^{t+1},y^{t+1})}{D_{o}{}^{t+1}(x^{t},y^{t})}\times \frac{D_{o}{}^{t}(x^{t+1},y^{t+1})}{D_{o}{}^{t}(x^{t},y^{t})}\right)}^{1/2}=\frac{D_{o}{}^{t+1}(x^{t+1},y^{t+1})}{D_{o}{}^{t}(x^{t},y^{t})}\times {\left(\frac{D_{o}{}^{t}(x^{t},y^{t})}{D_{o}{}^{t+1}(x^{t},y^{t})}\times \frac{D_{o}{}^{t}(x^{t+1},y^{t+1})}{D_{o}{}^{t+1}(x^{t+1},y^{t+1})}\right)}^{1/2}\\ ={\mathrm{EC}}^{t,t+1}\times {\mathrm{TC}}^{t.t+1} \end{array}$$

Furthermore, Färe and Grosskopf [32] decomposed the TC index into three separate indices including output-biased technical change (OBTC), input-biased technical change (IBTC) and the magnitude of technical change (MATC). These indices can disclose the tilted effects of the production frontier, measuring the contribution of three types of technical change including output-biased technical change, input-biased technical change and neutral technical change to the overall technical change and productivity growth. The three indices take the following forms:

(3) $$\begin{array}{l} \mathrm{OBTC}=\sqrt{\frac{D_{o}{}^{t}(x^{t+1},y^{t+1})}{D_{o}{}^{t+1}(x^{t+1},y^{t+1})}\times \frac{D_{o}{}^{t+1}(x^{t+1},y^{t})}{D_{o}{}^{t}(x^{t+1},y^{t})}};\;\\ \mathrm{IBTC}=\sqrt{\frac{D_{o}{}^{t}(x^{t+1},y^{t})}{D_{o}{}^{t+1}(x^{t+1},y^{t})}\times \frac{D_{o}{}^{t+1}(x^{t},y^{t})}{D_{o}{}^{t}(x^{t},y^{t})}}\\ \mathrm{MATC}=\frac{D_{o}{}^{t}(x^{t},y^{t})}{D_{o}{}^{t+1}(x^{t},y^{t})} \end{array}$$

If OBTC > 1, it means that the bias of output promotes technical progress and productivity growth, otherwise it leads to technical regress and productivity decline. OBTC = 1 means that there is no bias among outputs. If IBTC < 1, it indicates that the bias of input promotes technical progress and productivity growth, otherwise it leads to technical regress and productivity decline. IBTC = 1 indicates that there is no bias among inputs; MATC represents the magnitude of Hicks’ neutral technical progress. MATC > 1 means that the neutral technology promotes technical progress, and vice versa leads to technical regress. The Meanings of OBTC, IBTC and MATC are shown in Table 1.

**Figure 3:**
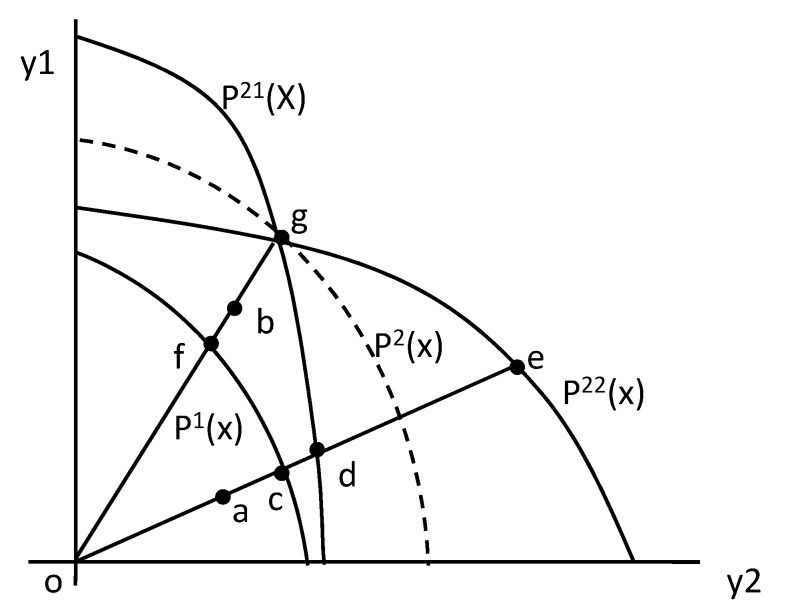
Output-biased technical change.

**Figure 4:**
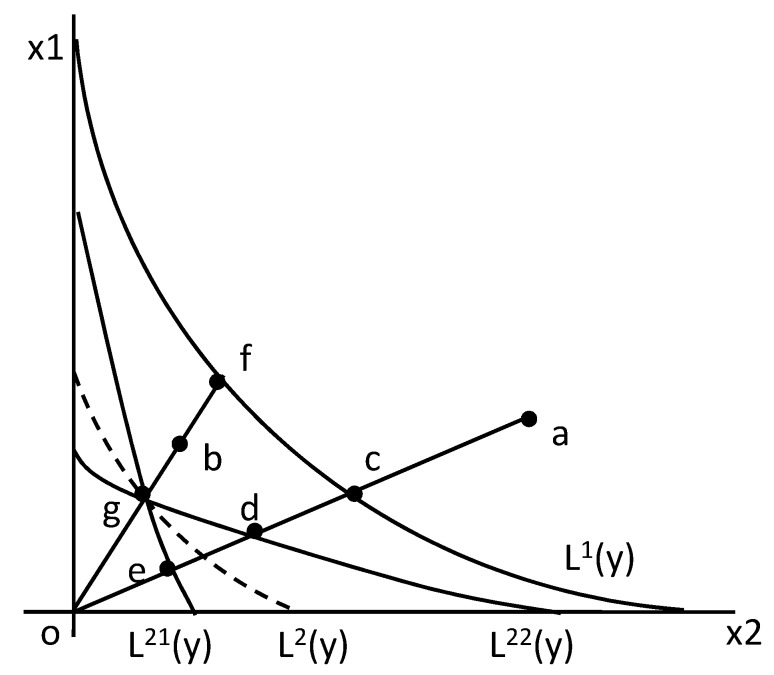
Input-biased technical change.

However, the indicator of OBTC or IBTC can only disclose whether the outputs or inputs are biased and whether the bias promotes technical progress, it can not directly indicate which output or input is biased. Weber and Domazlicky [33] and Barros and Weber [35] illustrate how to identify the directions of input bias and output bias in the biased technical change. In their analysis, the identifying criteria for biased technical change are on the ground of the input-based Malmquist productivity index. Färe et al. [36] demonstrate the criteria for identifying the direction of input bias in the biased technical change with the output-based Malmquist productivity index. We illustrate how to identify the directions of both the output and input bias in the biased technical change with the output-based Malmquist productivity index, then employing the identifying criteria to analyze the industrial green biased technical change in terms of water resources in China’s Yangtze River Economic Belt. The identifying criteria are shown in Table 2, which is derived from Figure 3 and Figure 4.

Figure 3 illustrates the construction of the output-biased technical change index. The production possibility frontier in period t is represented as P^t^(x), the inputs x can produce the outputs y, which include y_1_ and y_2_. We assumed there exists technical progress from period 1 to period 2. Technical progress is Hicks’ neutral if the MRT_y2y1_ (marginal rate of transformation) between two outputs of y_1_ and y_2_ remains constant. In this scenario, the production possibility frontier P^1^(x) in period 1 will move in parallel to P^2^(x) in period 2. If the technical progress leads to an output bias, the production possibility frontier will tilt when moving from period 1 to period 2. According to Weber and Domazlicky [33] and Barros and Weber [35], if the MRT_y2y1_ increases, the production possibility frontier in period 2 will be P^21^(x), indicating that the technical progress is biased towards y_1_, or y_1_-producing biased technical change. Otherwise, if MRT_y2y1_ decreases, the production possibility frontier in period 2 will be P^22^(x), indicating that the technical progress is biased towards y_2_, or y_2_-producing biased technical change. Now suppose that the output mix (y_1_, y_2_)^t^ for a DMU is at point “a” in period 1 and at point “b” in period 2, that means (y1y2)t=2>(y1y2)t=1, and suppose that the production possibility frontier is P^1^(x) in period 1 and P^21^(x) in period 2 which means there exists y_1_-producing biased technical change. In this situation, the Shephard output distance function in period 1 is $D_{o}{}^{1}(x^{},y^{1})=\mathrm{oa}/\mathrm{oc}$, and in period 2 is $D_{o}{}^{2}(x^{},y^{2})=\mathrm{ob}/\mathrm{og}$. The two inter-period input distance functions are calculated as $D_{o}{}^{2}(x^{},y^{1})=\mathrm{oa}/\mathrm{od}$ and $D_{o}{}^{1}(x^{},y^{2})=\mathrm{ob}/\mathrm{of}$, then according to the Formula (3), the OBTC index of DMU can be calculated as $\mathrm{OBTC}=\sqrt{\frac{ob/of}{ob/og}\times \frac{\mathrm{oa}/\mathrm{od}}{\mathrm{oa}/\mathrm{oc}}}=\sqrt{\frac{og/of}{od/oc}}>1$, so given that OBTC>1 and (y1y2)t=2>(y1y2)t=1, the technical change is biased towards y_1_. This is in the first situation. In the second situation, suppose that the outputs mix (y_1_, y_2_)^t^ for the DMU in each period and the production possibility frontier P^1^(x) in period 1 stay the same as the first situation, but the production possibility frontier in period 2 shifts to P^22^(x), which means there exists y_2_-producing biased technical change. In this situation, the OBTC index of DMU can be calculated as $\mathrm{OBTC}=\sqrt{\frac{ob/of}{ob/\mathrm{og}}\times \frac{\mathrm{oa}/\mathrm{oe}}{\mathrm{oa}/\mathrm{oc}}}=\sqrt{\frac{og/of}{oe/oc}}<1$, so given that OBTC<1 and (y1y2)t=2>(y1y2)t=1, the technical change is biased towards y_2_. In the third situation, if the production possibility frontier is P^2^ (x) in period 2, then the OBTC=1, so given that OBTC=1, technical change is neutral. The identifying criteria for output bias above are based on (y1y2)t=2>(y1y2)t=1, or the output y_1_ is increasing compared to y_2_ with time, which means the position of axis “ob” in period 2 is above the position of axis “oa” in period 1 as displayed in Figure 3. Oppositely, if (y1y2)t=2<(y1y2)t=1, the position of axis “ob” in period 2 would be underneath the position of axis “oa” in period 1. By exchanging the angle of axis “oa” and axis “ob” in Figure 3, it is not difficult to work out the identifying criteria on the condition of (y1y2)t=2<(y1y2)t=1, which are opposite to those on the condition of (y1y2)t=2>(y1y2)t=1. All the possible identifying criteria for the output-biased technical change are summarized in the left part of Table 2.

To investigate the input-biased technical change displayed in Figure 4, we rewrote the IBTC index in Formula (3) by the Shephard input distance function. Under the condition of constant returns to scale, the Shephard input distance function equals the reciprocal of the Shephard output distance function [43]. That is, $D_{I}{}^{t}(x_{k}{}^{t},y_{k}{}^{t})=D_{O}{}^{t}{\left(x_{k}{}^{t},y_{k}{}^{t}\right)}^{-1}$. Therefore, given constant returns to scale we can rewrite the IBTC index in Formula (3) as

(4) $$\mathrm{IBTC}=\sqrt{\frac{D_{I}{}^{t+1}(x^{t+1},y^{t})}{D_{I}{}^{t}(x^{t+1},y^{t})}\times \frac{D_{I}{}^{t}(x^{t},y^{t})}{D_{I}{}^{t+1}(x^{t},y^{t})}}$$

Figure 4 illustrates the construction of input-biased technical change index. The isoquant curve in period t is represented as L^t^(y), the inputs x can produce the outputs y. There are two kinds of inputs including x_1_ and *x*_2_. We assumed there exists technical progress from period 1 to period 2. If the technical progress is Hicks’ neutral, or the MRS_x2x1_ (marginal rate of substitution) remains constant, the isoquant curve L^1^(y) in period 1 will move in parallel to L^2^(y) in period 2. If the technical progress leads to input bias, the isoquant curve will tilt when moving from period 1 to period 2. If the MRS_x2x1_ increases, the isoquant curve in period 2 will be L^21^(y), indicating that the technical progress is biased towards saving *x*_1_, or *x*_2_-using biased technical change. Otherwise, if MRS_x2x1_ decreases, the isoquant curve in period 2 will be L^22^(y), indicating that the technical progress is biased towards saving x_2_, or x_1_-using biased technical change. Now suppose that the input mix (x_1_, *x*_2_)*^t^* for a DMU is at point “a” in period 1 and at point “b” in period 2, which means (x1x2)t=2>(x1x2)t=1, and suppose that the isoquant curve is L^1^(y) in period 1 and L^21^(y) in period 2, which mean there exists x_2_-using or x_1_-saving biased technical change. In this situation, The Shephard input distance function in period 1 is $D_{I}{}^{1}(x^{1},y)=\mathrm{oa}/\mathrm{oc}$, and in period 2 is $D_{I}{}^{2}(x^{2},y)=\mathrm{ob}/\mathrm{og}$. The two inter-period input distance functions are calculated as $D_{I}{}^{2}(x^{1},y)=\mathrm{oa}/\mathrm{oe}$ and $D_{I}{}^{1}(x^{2},y)=\mathrm{ob}/\mathrm{of}$. Then according to the Formula (4), the IBTC of the DMU can be calculated as $\mathrm{IBTC}=\sqrt{\frac{ob/og}{ob/of}\times \frac{oa/\mathrm{oc}}{oa/\mathrm{oe}}}=\sqrt{\frac{of/og}{oc/oe}}<1$. So, given that IBTC<1 and (x1x2)t=2>(x1x2)t=1, the technical change is biased towards x_2_-using or x_1_-saving. This is in the first situation. In the second situation, suppose that the input mix (x_1_, x_2_)^t^ for the DMU in each period and the isoquant curve L^1^(y) in period 1 stay the same as the first situation, but the isoquant curve in period 2 shifts to L^22^(y), which means there exists x_1_-using or x_2_-saving biased technical change. In this situation, IBTC of the DMU can be calculated as $\mathrm{IBTC}=\sqrt{\frac{ob/\mathrm{og}}{ob/of}\times \frac{\mathrm{oa}/\mathrm{oc}}{\mathrm{oa}/od}}=\sqrt{\frac{of/og}{oc/od}}>1$, so given that IBTC>1 and (x1x2)t=2>(x1x2)t=1, the technical change is biased towards x_1_-using or x_2_-saving. In the third situation, if the isoquant curve is L^2^ (y) in period 2, then OBTC=1, so give that IBTC=1, the technical change is neutral. The identifying criteria above for input bias are based on the condition of (x1x2)t=2>(x1x2)t=1, or the input ratio of x_1_ and x_2_ is increasing with time, which means the position of axis “ob” in period 2 is above the position of axis “oa” in period 1 as displayed in Figure 4. Oppositely, if (x1x2)t=2<(x1x2)t=1, the position of axis “ob” in period 2 would be underneath the position of “oa” in period 1. By exchanging the angle of axis “oa” and axis “ob” in Figure 4, it is not difficult to work out the identifying criteria for input bias on the condition of (x1x2)t=2<(x1x2)t=1, which are opposite to those on the condition of (x1x2)t=2>(x1x2)t=1. All the possible identifying criteria for the input-biased technical change are summarized in the right part of Table 2.

It should be noted that the identifying criteria for biased technical change in Table 2 are derived from the output-based Malmquist productivity index as by Fare et al. [36]. The results are a little different to those of Weber and Domazlicky [33] and Barros and Weber [35]. The criteria for biased technical change constructed by Weber and Domazlicky [33] and Barros and Weber [35] are derived from the input-based Malmquist productivity index, which is the reciprocal of output-based Malmquist productivity index given constant returns to scale. Therefore, in our research, OBTC > 1 or IBTC >1 indicates a biased technical progress rather than biased technical regress as in their research. After controlling this difference, the identifying criteria of Table 2 is substantially consistent with those of Weber and Domazlicky [33] and Barros and Weber [35].

The economic meaning for the identifying criteria of output-biased technical change in Table 2 can be well explained. Under the y_1_-producing biased technical change mode, only if the growth rate of output y_1_ from period 1 to period 2 is higher than that of output y_2_ can the productivity be promoted by the output-biased technical change (OBTC > 1), otherwise, the productivity will decrease (OBTC < 1). In short, the structure of output mix needs to match the inner request of the output-biased technical change in order to promote the productivity. The economic meaning is similar for the identifying criteria of input-biased technical change. Under the x_1_-using or *x*_2_-saving technical change mode, only if the saving speed of input x_2_ from period 1 to period 2 is faster than that of input x_1_ can the productivity be promoted by the input-biased technical change (IBTC > 1), otherwise the productivity will decrease (IBTC < 1). In short, the structure of input mix needs to match the inner request of the input-biased technical change in order to promote the productivity.

### 3.2. Indicators and Data

In our research, two output indicators were considered including the industrial added value and COD clean index.

Industrial added value: The industrial added value represents the economic output of the industrial economic development. It is measured by the added value of industrial enterprises above the designated size in each provincial region across China. It is deflated by the deflator of the industrial producer price index and denoted at the price of 2005.

COD clean index: COD clean index is constructed to measure water conservation performance, which is related to the COD emission (chemical oxygen demand) among industrial sewage in provincial regions across China. Since COD emission is an undesirable or negative output, it is necessary to transform the negative index to a positive index in order to use the Shephard radial distance function and the identifying criteria of biased technical change in Table 2, which is derived on the assumption of Shephard radial distance function. One main method for transforming a negative indicator to a positive indicator is to transform the negative indicator to the form of additive inverses (-y), and add to the additive inverses a sufficient large positive constant c, then construct the transformed positive indictor y’ by y’= −y + c [44,45]. The advantage of this transformation method is that it does not change the internal linear structure of the original data. In order to ensure that y’ is positive, we let c = y_max_ + y_min_, where y represents the COD emission of each DMU. With y’ = −y + c, the maximum value of original COD emission is reversed to the minimum value, and the original minimum is reversed to the maximum. After that, the COD clean index is constructed by (y’/y’_min_) × 100. The COD clean index is the desirable or positive output index. The larger the COD clean index, the cleaner the industrial sewage.

There are three input indicators, including capital stock, labor and water consumption.

Capital stock: Capital stock is measured by the stock of physical assets investment of industrial enterprises above the designated size in provincial regions across China, calculated by the perpetual inventory method [46] as $K_{it}=I_{it}+(1-\delta )K_{i,t-1}$, where *K_it_* represents capital stock of region *i* in period *t*, *I_it_* represents capital flow of region *i* in period *t*, δ represents the economic depreciation rate. The initial capital stock in 2005 is presented by the net value of physical assets in 2005. As to the economic depreciation rate δ, we assumed a value of 11.6%, which is estimated by Shan et al. [47] and has been widely adopted in estimating the capital stock in China. The capital stock is deflated by the deflator of the capital investment price index at the price of 2005.

Labor: Labor represents the input of human resources in industrial production. It is represented by the annual average number of employees in industrial enterprises above designated size at provincial level across China.

Water consumption: Water consumption represents the input of water in industrial production. It is measured by the industrial water consumption of industrial enterprises above the designated size at the provincial level across China.

The data for the industrial index, such as the industrial added value, physical assets investment and industrial labor are extracted from China Industry Statistical Yearbook from 2005 to 2015 and the related provincial statistical yearbooks in China. The data for environmental index, such as the water consumption and the COD emission among industrial sewage are taken from China Environmental Statistical Yearbook from 2005 to 2015. The price index is from the China Statistical Yearbook from 2005 to 2015.

## 4. Results and Discussion

### 4.1. The Descriptive Statistics of Variables

The study employed relevant input–output data for the period 2005–2015 to calculate the green productivity change index and decomposition index from 2006 to 2015. The left side of Table 3 presents the descriptive statistics from 30 provincial regions during 2005 to 2015 across mainland China (except for Tibet due to incomplete data), the right side of Table 3 presents the descriptive statistics from 11 provincial regions in YREB. According to Table 3, the provincial average water consumption level in YREB was higher than that nationwide in China, and the provincial average COD Clean Index level in YREB was similar to that nationwide.

### 4.2. Industrial Green Productivity Change in Terms of Water Resources in YREB

Firstly, the DEA scores that represent the green productivity in terms of water resources in each province of YREB during 2006–2015 were estimated. These estimates in YREB represent the relative productivity level compared to the production frontier nationwide in China. Then, based on these estimates, the MI (Malmquist index) in YREB as well as the decomposition index during the period of 2006–2015 were estimated. According to Table 4, the average DEA score during 2006–2015 was 0.72, which means that given the input level, the industrial green output level in YREB was 72% of the potentially maximum output level in the production frontier nationwide, and there was still a space of 28% for the green productivity improvement for YREB. During 2006–2015, the average MI index was greater than 1, indicating that the green productivity of water resources in YREB was generally increasing. From the perspective of the MI’s decomposition index, the average TC index was greater than 1 during 2006–2015, indicating that during 2006–2015, the technical change generally promoted the green productivity of industrial water resources in YREB. According to the decomposition index of TC, the average values of OTBC (output-biased technical changes index), ITBC (input-biased technical change index) and MATC (neutral technical change index) were all greater than 1 from 2006 to 2015, indicating that all three types of technical change exist simultaneously. Although the neutral technical change had a greater impact on the productivity, technical change had also experienced an output bias and input bias, and these biases generally promoted the productivity during 2006–2015.

**Figure 5:**
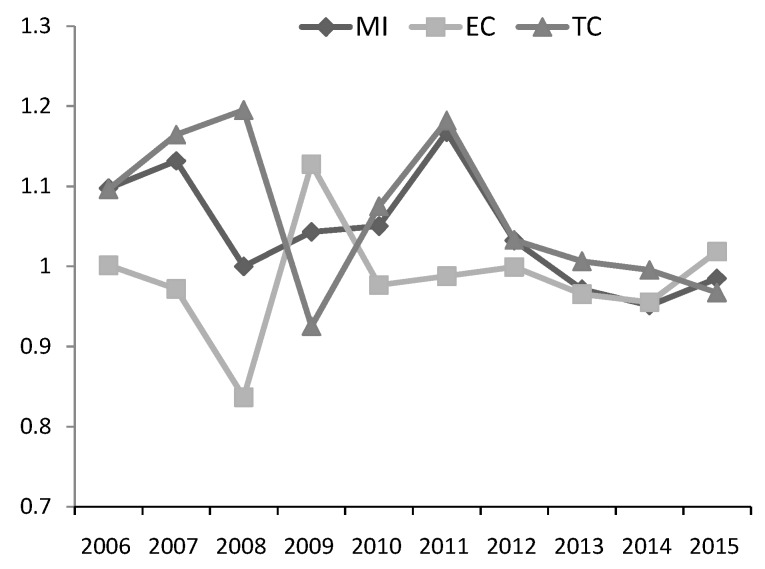
Annual MI, efficiency change (EC) and technical change (TC) index.

**Figure 6:**
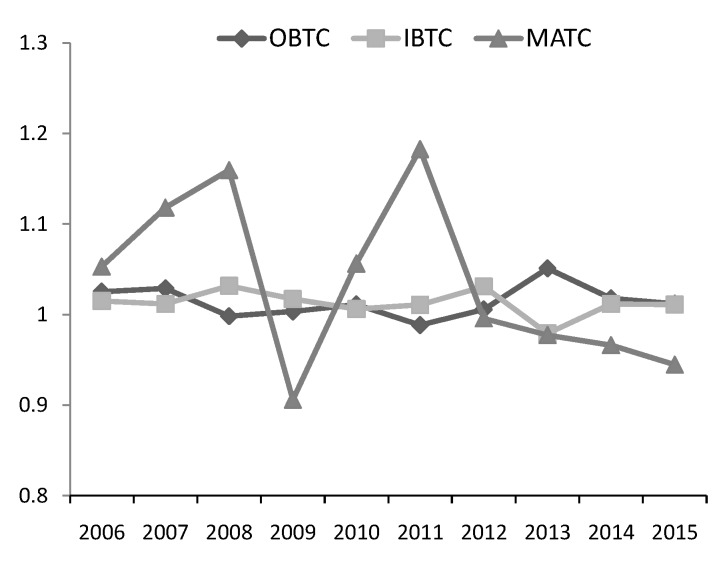
Annual biased technical change index.

Figure 5 and Figure 6 show the dynamics of the MI index and the decomposition index during 2006–2015. According to Figure 5, although the MI index was fluctuating during 2006–2015, it was greater than 1 in most years, indicating that the green productivity was increasing in most years since 2006. TC index was greater than 1 in most years, but the growth rate was slowing down after 2011. The trend of TC index was similar to that of MI index, which means the technical change was the main factor affecting productivity change.

Figure 6 shows the dynamics of biased technical change index, including OBTC, IBTC and MATC. According to Figure 6, although less fluctuating compared to the MATC index, the OBTC index and the IBTC index all present the volatility, and they were greater than 1 in most years from 2006 to 2015. Figure 6 revealed consistent findings with Table 4. Although the neutral technical change had a greater impact on the productivity change, the technical change had also experienced the input and output bias simultaneously, and the biased technical change had increased the productivity in most years from 2006 to 2015.

### 4.3. Output-Biased Technical Change

The green biased technical change in terms of industrial water resources at the output side in YREB was discussed in this section. Firstly, based on the identifying criteria for output-biased technical change in Table 1, the numbers of provincial regions in YREB that experience y_i_-producing biased technical change were sorted out each year from 2006 to 2015. Then, taking two years as a statistical period, the proportions of provincial regions in YREB that experience y_i_-producing biased technical change were calculated. In addition, the overall proportions that experience y_i_-producing biased technical change from 2006 to 2015 were also calculated. The overall results of output-biased technical change are presented in Table 5. In Table 5, y_1_ represents the industrial GDP, y_2_ represents the COD clean index. The larger the COD clean index, the higher the water conservation performance. Technical change biased to y_1_ indicates that under this kind of technical change mode, focusing more on economic growth than water protection can promote productivity (OBTC > 1), otherwise the productivity will decline (OBTC < 1). This kind of output-biased technical change (y_1_-producing) can be called economy-growth biased technical change. Under the economy-growth biased technical change, the growth of green productivity is mainly driven by economic performance rather than water protection performance. On the other hand, technical change biased to y_2_ means that under this kind of technical change mode, focusing more on water protection performance than economic growth can promote productivity (OBTC > 1), otherwise the productivity will decline (OBTC < 1). This kind of output-biased technical change (y_2_-producing) can be called water-conservation biased technical change. Under the water-conservation biased technical change, the growth of green productivity is mainly driven by water protection performance rather than economic performance.

According to Table 5, there were three main findings. Firstly, the proportion of regions in YREB with the economy-growth biased technical change was higher than that with the water-conservation biased technical change during the period of 2006–2015 as a whole. The total number of regions with the economy-growth biased technical change accounted for 46% of all the regions during 2006–2015, while the total number of regions experiencing water-conservation biased technical change only accounted for 28%. Secondly, the proportion of regions with OBTC > 1 was more than that with OBTC < 1 in most two-year periods, which means the output-biased technical change (including both two types of output-biased technical change) was generally boosting the productivity in YREB. Finally, within the regions where OBTC > 1, the proportion of regions with economy-growth biased technical change was larger than those with the water-conservation biased technical change in each two-year period, which means the economy-growth biased technical change is the main force to promote productivity, whereas the role of water-conservation biased technical change was insufficient. Green technology innovations in industrial sewage control and treatment need to catch up with the economic growth in YREB.

**Figure 7:**
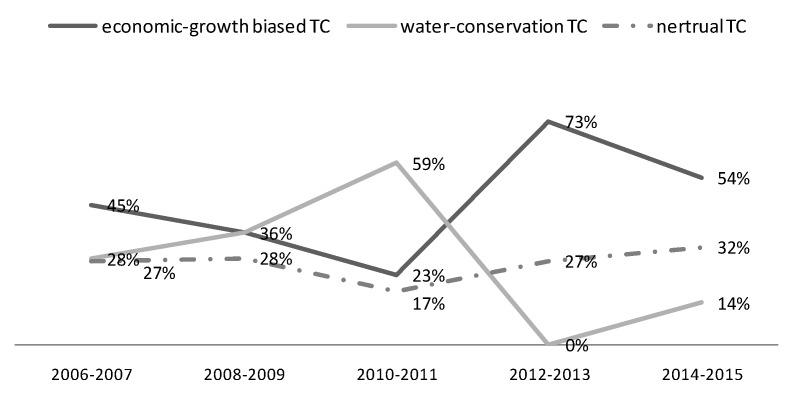
The dynamics of output-biased technical change.

Figure 7 reveals the dynamics of output-biased technical change in the YREB from 2006 to 2015. In general, the proportion of water-conservation biased technical change and economy-growth biased technical change both present great fluctuations. Since 2008, the proportion of regions in YREB experiencing water-conservation technical change increased rapidly, but it declined in the period of 2012–2013, and then increased again. As far as the regulations are concerned, with the implementation of the “ Amendment of China Water Pollution Prevention and Control Law” in 2008, the water-conservation biased technical change was promoted due to more concerns about the water protection performance in YREB, but the impact of new regulation was limited with time. After 2012, policies such as the “Opinions on Implementing the Strictest Water Resources Management System” and the “Water Pollution Prevention and Control Action Plan” once again promoted the water-conversation biased technical change. So, the trend of water-conversation biased technical change in YREB was in line with the release time of water-related environmental policies and regulations from 2008, which indicates that China’s water-related environmental policies and regulations from 2008 had positively influenced the propensity of water-conversation biased technical change in YREB. However, in general, due to the great pressure of GDP growth, the economy-growth biased technical change mode is more prevalent than the water-conservation biased technical change model after 2011.

### 4.4. Input-Biased Technical Change

In this section, the green biased technical change in terms of industrial water resources at the input side in YREB is discussed based on the identifying criteria for input-biased technical change in Table 1. Table 6 shows the proportion of provincial regions in YREB that experience x_i_-using (or x_j_-saving) biased technical change during 2006–2015 by pairwise comparison of three input factors including industrial water consumption (x_1_), capital (x_2_) and labor (x_3_). Technical change biased towards x_i_ indicates that under this kind of technical change mode, relatively more use of x_i_ and saving x_j_ could increase productivity (IBTECH > 1), otherwise the productivity would decline (IBTECH < 1).

(1) As to the pair of water consumption (x_1_) and capital (x_2_), according to Table 6, the total number of regions experiencing capital-using biased technical change accounted for 46% of all the regions during 2006–2015, whereas the total number of regions experiencing water-using biased technical change only accounted for 39%. So, taking the 10 years as a whole, most of the regions experience the capital-using biased technical change rather than the water-using biased technical change. Furthermore, within regions where IBTC > 1, the proportion of regions with capital-using biased technical change was larger than those with water-conservation biased technical change in each two-year period, which means the capital-using or water-saving biased technical change is the dominant force to promote productivity from 2006 to 2015. Since 2008, the Chinese government has issued a series of important water protection policies. The cost of industrial wastewater treatment and penalty has been increasing, accordingly raising the cost of industrial water consumption. As a result, more and more provinces in YREB have begun developing or adopting new technologies to use more capital and save water. This has promoted the capital-using biased technical change or water-saving biased technical change, as well as the green productivity in YREB.

**Figure 8:**
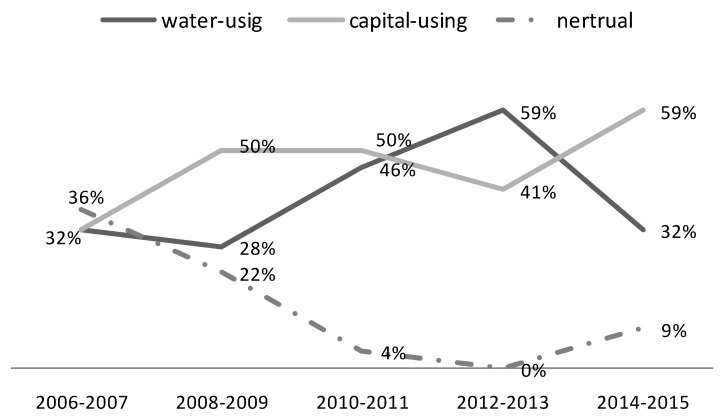
The dynamics of input-biased technical change as to the pair of water consumption and capital.

Figure 8 reveals the dynamics of input-biased technical change regarding the pair of water consumption and capital during the period 2006–2015. Generally, except for the period of 2012 to 2013, the capital-using technical biased change mode is dominated compared to the water-using technical biased change mode. Yet the capital-using biased technical change mode is not as popular as before from 2010 to 2013. The global financial crisis since 2008 has a greater impact on China’s foreign trade industry, leading to a decline in the marginal output of capital for several years [48]. Although the costs related to industrial water use are also increasing due to more strict water regulation since 2008, the relative attractiveness of capital factor is still insufficient compared to other factors such as water. So, the capital-biased technical biased change mode in YREB lacks attractiveness for several years after 2010. However, after 2013, with water regulation becoming more stringent in China, and the global economy gradually recovering from the global financial crisis, the capital-biased technical biased change mode regains its popularity in YREB.

(2) As to the pair of water consumption (x_1_) and labor (x_3_), according to Table 6, the total number of regions experiencing water-using biased technical change accounted for 45% of all the regions during 2006–2015, and the total number of regions experiencing labor-using biased technical change accounted for 40%. So, taking 10 years as a whole, the water-using biased technical change was slightly dominant compared to the labor-using biased technical change.

**Figure 9:**
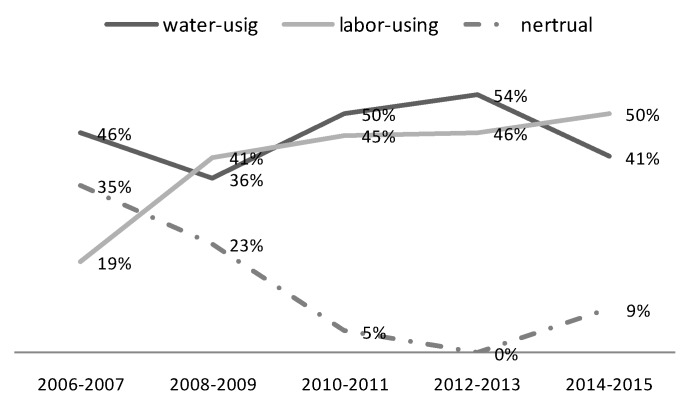
The dynamics of input-biased technical change as to the pair of water consumption and labor.

However, according to Figure 9, the dynamic analysis shows that the proportion of regions experiencing labor-using biased technical change was catching up from 2008. With the increasingly rigorous water regulation, the cost of industrial water was not as cheap as before, the cost advantage of water factor over labor factor in the industrial production was decreasing, more enterprises were seeking technologies to use more labor and save water. Therefore, the labor-using biased or water-saving biased technical change was catching up from 2008 to 2015 in YREB.

Furthermore, within regions where IBTC > 1, the proportion of regions with labor-using biased technical change was larger than those with water-using biased technical change in each two-year period from 2008, which means the labor-using or water-saving biased technical change was becoming the dominant force to promote productivity from 2008 to 2015.

(3) In terms of capital (x_2_) and labor (x_3_), according to Table 6, the proportion of regions experiencing capital-using biased technical change accounted for 47% of all the regions during 2006–2015, whereas the proportion of regions experiencing labor-using biased technical change accounted for 38%. So, during 2006–2015, the industrial technical change was generally biased towards capital compared to labor in YREB area. The results were consistent with the existing research, which indicate that Chinese industrial technical change is apparently biased towards capital compared with labor since the 1990s [49].

## 5. Conclusions

The Yangtze River Economical Belt (YREB) is a significant ecological corridor from west to east across China. Although the economic performance and green performance are both important in YREB, the dominance between them varies in different regions and at different times. Rather than only focusing on the overall growth of green productivity, it is necessary to identify whether the technical change is biased towards economic performance or green performance in promoting the green productivity in YREB. Using biased technical change theory and Malmquist index decomposition method, we analyzed the green biased technical change in terms of industrial water resources in YREB at the output side and the input side respectively. We found that the green biased technical change varied during 2006–2015 at both sides in YREB. At the output side, the output-biased technical change in YREB shows that the technical change was more biased to economic growth than water resources protection in YREB. The economy-growth biased technical change was the main force to promote green productivity, whereas the role of water-conservation biased technical change was insufficient. The green performance at the output side needs to catch up with the economic performance in YREB. As to the input side and the input-biased technical change, the pair comparison of capital and water shows that a majority of regions in YREB experience capital-using or water-saving biased technical change during the period of 2006–2015. With respect to the pair comparison of labor and water, although the water-using biased technical change was slightly dominant during the period of 2006–2015, the labor-using or water-saving biased technical change was catching up from 2008. Combining the analysis of both the input bias and the output bias of technical change, it is shown that a series of water environmental policies introduced in China since 2008 had begun to impact the input side of the industrial production with water-saving effects in YREB, but the policy effects were not enough from the perspective of the output side. The technological innovation in terms of industrial sewage control and treatment need to catch up with the economical growth.

The green technological innovation and green productivity were becoming the main driver for the sustainable development in China. Green biased technical change should be strengthened in the YREB in order to let the green performance catch up with the economic performance in YREB. Some regions with severe water pollution should focus on water-conservation biased technical change, which means that they can achieve the simultaneous development of environmental protection and economic growth while putting water environmental protection first. Both science and technology policies and environmental policies should be employed. In terms of the science and technology policies, university-industry R&D cooperation in the field of green technology should be encouraged. Consistently strengthening intellectual property protection on green technology is also important. As to the environmental policies, more stringent legislation and enforcement related to environmental protection is necessary to incentivize the creation and adoption of green technological innovation. By these policies and activities, green biased technical change can be significantly advanced to implement the green transformation of industrial economy in China’s Yangtze River Economic Belt.

## Figures and Tables

**Table 1:** Output-biased technical change (OBTC), input-biased technical change (IBTC) and magnitude of technical change (MATC).

Index	>1	<1	=1
OBTC	Output-biased technical progress	Output-biased technical regress	No output-biased technical change
IBTC	Input-biased technical progress	Input-biased technical regress	No input-biased technical change
MATC	Neutral technical progress	Neutral technical regress	No neutral technical change

**Table 2:** Identifying criteria for biased technical change.

Output-Biased Technical Change			Input-Biased Technical Change		
Output Mix	OBTC>1	OBTC<1	Input Mix	IBTC>1	IBTC<1
(y1y2)t=2>(y1y2)t=1	*y_1_-producing*	*y_2_-producing*	(x1x2)t=2>(x1x2)t=1	*x_1_-using, or x_2_-saving*	*x_2_-using*, *or x_1_-saving*
(y1y2)t=2<(y1y2)t=1	*y_2_-producing*	*y_1_-producing*	(x1x2)t=2<(x1x2)t=1	*x_2_-using, or x_1_-saving*	*x_1_-using, or x_2_-saving*

**Table 3:** Descriptive statistics of variables (2005–2015).

Stage	Variable	National					YREB ^a^				
		Mean	Std. Dev.	Max	Min	*N*	Mean	Std. Dev.	Max	Min	*N*
Input	Water (B Ton)	4.604	4.501	23.900	0.240	330	7.561	4.986	23.900	1.838	121
	Capital (B CNY)	552.562	496.809	3042.125	29.729	330	588.161	512.524	3042.125	110.012	121
	Labor (M People)	2.955	3.226	15.680	0.116	330	3.324	2.867	11.539	0.666	121
Output	Industrial GDP (B CNY)	569.279	609.286	3195.246	15.250	330	594.690	552.280	2916.019	58.585	121
	COD Clean Index	11528	2205	14341	100	330	11546	1714	14167	7312	121

^a^ YREB means the Yangtze River Economic Belt.

**Table 4:** Data envelopment analysis (DEA), Malmquist index (MI) and the decomposition index.

Year	DEA	MI	EC	TC	OBTC	IBTC	MATC
2006–2010	0.741	1.064	0.978	1.087	1.013	1.017	1.055
2011–2015	0.699	1.019	0.985	1.034	1.015	1.009	1.010
2006–2015	0.720	1.041	0.982	1.060	1.014	1.013	1.032

**Table 5:** Output-biased technical change (%) ^a^.

		(y1y2)t+1>(y1y2)t	(y1y2)t+1<(y1y2)t			(y1y2)t+1>(y1y2)t	(y1y2)t+1<(y1y2)t
2006–2007	OBTECH > 1	45 (y_1_-producing)	0 (y_2_-producing)	2012–2013	OBTECH>1	73 (y_1_-producing)	0 (y_2_-producing)
	OBTECH < 1	28 (y_2_-producing)	0 (y_1_-producing)		OBTECH < 1	0 (y_2_-producing)	0 (y_1_-producing)
	Neutral	27			Neutral	27	
2008–2009	OBTECH > 1	36 (y_1_-producing)	0 (y_2_-producing)	2014–2015	OBTECH > 1	36 (y_1_-producing)	14 (y_2_-producing)
	OBTECH < 1	36 (y_2_-producing)	0 (y_1_-producing)		OBTECH < 1	0 (y_2_-producing)	18 (y_1_-producing)
	Neutral	28			Neutral	32	
2010–2011	OBTECH > 1	23 (y_1_-producing)	9 (y_2_-producing)	**2006–2015**		**y_1_-producing**	**46**
	OBTECH < 1	50 (y_2_-producing)	0 (y_1_-producing)			**y_2_-producing**	**28**
	Neutral	18				**Neutral**	**26**

^a^ y_1_ represents the industrial GDP, y_2_ represents the COD clean index.

**Table 6:** Input-biased technical change (%) ^a^.

		(x1x2)t+1>(x1x2)t	(x1x2)t+1<(x1x2)t	(x1x3)t+1>(x1x3)t	(x1x3)t+1<(x1x3)t	(x2x3)t+1>(x2x3)t	(x2x3)t+1<(x2x3)t
2006–2007	IBTECH > 1	9 (x_1_-using)	18 (x_2_-using)	14(x_1_-using)	14 (x_3_-using)	27 (x_2_-using)	0 (x_3_-using)
	IBTECH < 1	14 (x_2_-using)	23 (x_1_-using)	5 (x_3_-using)	32 (x_1_-using)	18 (x_3_-using)	19 (x_2_-using)
	Neutral	36		35		36	
2008–2009	IBTECH > 1	5 (x_1_-using)	41(x_2_-using)	9 (x_1_-using)	36 (x_3_-using)	32 (x_2_-using)	14 (x_3_-using)
	IBTECH < 1	9 (x_2_-using)	23 (x_1_-using)	5 (x_3_-using)	27 (x_1_-using)	18 (x_3_-using)	14 (x_2_-using)
	Neutral	22		23		22	
2010–2011	IBTECH > 1	14(x_1_-using)	32 (x_2_-using)	18 (x_1_-using)	27(x_3_-using)	27 (x_2_-using)	27 (x_3_-using)
	IBTECH < 1	18 (x_2_-using)	32 (x_1_-using)	18 (x_3_-using)	32 (x_1_-using)	27 (x_3_-using)	14 (x_2_-using)
	Neutral	4		5		5	
2012–2013	IBTECH > 1	5 (x_1_-using)	36 (x_2_-using)	9 (x_1_-using)	32 (x_3_-using)	41 (x_2_-using)	14 (x_3_-using)
	IBTECH < 1	5(x_2_-using)	54 (x_1_-using)	14 (x_3_-using)	45 (x_1_-using)	36 (x_3_-using)	9(x_3_-using)
	Neutral	0		0		0	
2014–2015	IBTECH > 1	9 (x_1_-using)	59 (x_2_-using)	23 (x_1_-using)	45 (x_3_-using)	55 (x_2_-using)	14 (x_3_-using)
	IBTECH < 1	0 (x_2_-using)	23 (x_1_-using)	5 (x_3_-using)	18 (x_1_-using)	23(x_3_-using)	0 (x_3_-using)
	Neutral	9		9		8	
**2006–2015**		**x_1_-using**	**39**	**x_1_-using**	**45**	**x_2_-using**	**47**
		**x_2_-using**	**46**	**x_3_-using**	**40**	**x_3_-using**	**38**
		**Neutral**	**15**	**Neutral**	**15**	**Neutral**	**15**

^a^ x_1_ represents industrial water consumption, x_2_ represents capital, x_3_ represents labor.
